# A homozygous *KASH5* frameshift mutation causes diminished ovarian reserve, recurrent miscarriage, and non-obstructive azoospermia in humans

**DOI:** 10.3389/fendo.2023.1128362

**Published:** 2023-02-14

**Authors:** Xiaoning Hou, Aurang Zeb, Sobia Dil, Jianteng Zhou, Huan Zhang, Baolu Shi, Zubair Muhammad, Ihsan Khan, Qamar Zaman, Wasim Akbar Shah, Xiaohua Jiang, Limin Wu, Hui Ma, Qinghua Shi

**Affiliations:** ^1^ The First Affiliated Hospital of University of Science and Technology of China, Hefei, China; ^2^ School of Basic Medical Sciences, Division of Life Sciences and Medicine, University of Science and Technology of China, Hefei, China; ^3^ Biomedical Sciences and Health Laboratory of Anhui Province, Hefei, China; ^4^ Institute of Health and Medicine, Hefei Comprehensive National Science Center, Hefei, China

**Keywords:** LINC complex, KASH5, meiotic arrest, diminished ovarian reserve (DOR), non-obstructive azoospermia (NOA), recurrent miscarriage (RM)

## Abstract

The meiosis-specific LINC complex, composed of the KASH5 and SUN1 proteins, tethers the moving chromosomes to the nuclear envelope to facilitate homolog pairing and is essential for gametogenesis. Here, we applied whole-exome sequencing for a consanguineous family with five siblings suffering from reproductive failure, and identified a homozygous frameshift mutation in *KASH5* (c.1270_1273del, p.Arg424Thrfs*20). This mutation leads to the absence of KASH5 protein expression in testes and non-obstructive azoospermia (NOA) due to meiotic arrest before the pachytene stage in the affected brother. The four sisters displayed diminished ovarian reserve (DOR), with one sister never being pregnant but still having dominant follicle at 35 years old and three sisters suffering from at least 3 miscarriages occurring within the third month of gestation. The truncated KASH5 mutant protein, when expressed in cultured cells, displays a similar localization encircling the nucleus and a weakened interaction with SUN1, as compared with the full-length KASH5 proteins, which provides a potential explanation for the phenotypes in the affected females. This study reported sexual dimorphism for influence of the *KASH5* mutation on human germ cell development, and extends the clinical manifestations associated with *KASH5* mutations, providing genetic basis for the molecular diagnosis of NOA, DOR, and recurrent miscarriage.

## Introduction

Reproductive failure is a global public health issue, affecting dozens of millions of couples in the world (1). It can be caused by male factor, female factor, or both. For failure attributed to the male side, non-obstructive azoospermia (NOA), characterized by absence of spermatozoa in the ejaculates due to spermatogenic failure, is the most severe type. For failure attributed to the female side, diminished ovarian reserve (DOR) and recurrent miscarriage are two of the major challenges of the reproductive medicine. DOR is defined as a decreased antral follicle count on ultrasound and/or abnormal serum testing (i.e., elevated FSH and/or low AMH levels) in the setting of regular menstrual cycles (2). It is estimated that 26% of the assisted reproductive technology population have DOR and the number is increasing (3). Recurrent miscarriage is defined as the loss of two or more consecutive clinical pregnancies before 20–24 weeks of gestation (4, 5). It affects ~2.5% of women trying to conceive and approximately half of them do not have a clearly defined etiology (5). Elucidating the underlying etiology could provide guidance on the clinical management and effective therapeutic interventions of the affected individuals.

Meiosis consists of two consecutive cell divisions by which sexual organisms produce haploid gametes from a diploid germ cell. During meiosis, homologous chromosomes pair, synapse, engage in recombination and then segregate to opposite poles at the first meiotic division, followed by separation of sister chromatids to opposite poles at the second division (6). The pairing of homologous chromosomes is driven by homology-directed repair of DNA double-strand breaks (DSBs), through which homologous chromosomes find each other and move close. Then a synaptonemal complex (SC) forms along the entire length of homologous chromosomes and mediates the synapsis of homologous regions. Notably, to facilitate homologous chromosome pair and synapsis, telomeres attach to the inner nuclear envelope and skate along the inner membrane until all chromosome ends cluster to form a meiosis-unique structure that resembles a bouquet of flowers (7).

The bouquet configuration is achieved by the linker of nucleoskeleton and cytoskeleton (LINC) complex that couples telomeres of meiotic chromosomes to cytoplasmic dynein, enabling dynamic movement of telomeres on the nuclear envelope (8). In mice, the LINC complex consists of two members: the SUN1, a SUN (Sad1, Unc-84) domain protein localizing on the inner nuclear envelope and KASH5, a KASH (Klarsicht, ANC-1, Syne Homology) domain protein localizing on the outer nuclear envelope (9–11). KASH5, also known as CCDC155, is the only KASH domain protein expressed in meiocytes (12). The N-terminus of KASH5 binds to cytoplasmic dynamin, while the C-terminus, comprising of a single-transmembrane region, extends into the perinuclear space to interact with SUN1, which binds to telomeres and is essential for the telomere localization of KASH5 (12, 13). In *Kash5*-deficient mice, telomeres could attach to nuclear envelope, but paring of homologous chromosomes fails and meiosis arrests prior to the pachytene stage, indicating a crucial role of KASH5 in coupling telomere to the cytoplasmic dynein to ensure the chromosome alignment (13). Very recently, homozygous splicing and missense mutations in *KASH5* were reported in NOA men displaying zygotene arrest and women with premature ovarian insufficiency (POI), suggesting a potentially conserved role of KASH5 in humans (14–18). However, the underlying pathogenic mechanism is not yet fully understood.

In this study, we identified a homozygous *KASH5* frameshift mutation in five siblings born to a consanguineous marriage. They have been married for at least 16 years and tried continually to conceive, but could not give birth to any children. The mutation leads to a complete absence of KASH5 proteins and NOA in the affected brother. Intriguingly, the four affected sisters displayed DOR, rather than POI. One sister has never been pregnant but still had dominant follicles at the age of 35, and the other three sisters experienced at least three miscarriages during the third month of gestation. Therefore, our results indicate sexual dimorphism in effects of the *KASH5* mutation on germ cell survival and development of humans and that KASH5 could be a potential diagnosis marker of DOR and recurrent miscarriage, in addition to NOA and POI previously reported.

## Materials and methods

### Clinical samples

This study reported five siblings, one brother and four sisters from a consanguineous Pakistani family (recorded at the Human Reproduction Case Bank of the University of Science and Technology of China), suffering from idiopathic reproductive failure. Written informed consent was received from all participants prior to the onset of the study. For the male patient, semen analyses were performed twice following the guidelines of World Health Organization (WHO) (19). He had testicular biopsy performed. The testicular tissue was fixed in 4% paraformaldehyde (PFA) and Bouin’s solution (Sigma, HT10132, Taufkirchen, Germany) and embedded into paraffin blocks for sectioning and subsequent histological and immunofluorescence staining analyses. The studies involving human participants were reviewed and approved by institutional ethics committee of the University of Science and Technology of China (USTC). The patients/participants provided their written informed consent to participate in this study.

### Whole exome sequencing, variant filtration and validation

Total genomic DNA was extracted from the peripheral blood of individuals using a FlexiGene DNA Kit (QIAGEN, 51206, Hilden, Germany). Whole-exome capture and sequencing were performed using the xGen EXOME RESEARCH PANEL V2 (Integrated DNA Technologies, Shanghai, China) and MGISEQ-2000 platform (BGI, China) following the standard procedures. Clean reads were aligned to the human genome reference assembly (hg19) using Burrows–Wheeler Aligner (BWA) with default parameters (20). Then, Picard software (http://picard.sourceforge.net/) was employed to remove polymerase chain reaction (PCR) duplicates. DNA sequence variants were called using the Genome Analysis Toolkit HaplotypeCaller (http://www.broadinstitute.org/gatk/). Variants were annotated using ANNOVAR (21).

Candidate pathogenic variant filtration was performed in a stepwise manner as we previously described (**Supplementary Figure 1**). Firstly, linkage analysis was performed using PedMiner and two regions were identified with logarithm of the odds scores >0.5(22) (**Supplementary Figure 1B**). Variants within linkage regions and following recessive inheritance were kept for further screening. Runs of homozygosity (RoH) were detected using BCFtools/RoH and RoH regions >1.5Mb were used to calculate the FROH value to measure the inbreeding coefficients using our inhouse scripts(23). Variants following recessive inheritance with infertility/recurrent miscarriage and meeting the following conditions were given preference: (1) variants potentially affecting protein sequence (nonsense, missense, splice-site variants, and coding indels); (2) variants with minor allele frequencies (MAF) <0.01 in 1000 Genomes project, ESP6500, or GnomAD database; (3) loss-of-function variants or potentially deleterious missense variants predicted by seven software including Sorting Intolerant From Tolerant (SIFT)(24), PolyPhen-2(25), and MutationTaster(26), *etc.*; (4) variants within genes expressed in testis. Secondly, variants following dominant inheritance with female infertility or recurrent miscarriage and meeting the following conditions were given preference: (1) variants potentially affecting protein sequence (nonsense, missense, splice-site variants, and coding indels); (2) variants heterozygous in our in-house fertile control females were excluded; (3) variants in genes for which the inactivation has no effect on female fertility were excluded. Finally, variants within genes for which the inactivation leads to reproduction defects resembling our patients based on MGI database(27), FertilityOnline database or literature search were considered in priority. The retained candidate pathogenic variant was subsequently verified by Sanger sequencing. Primer sequences used are shown in **Supplementary Table 2**.

### RT-PCR

Testicular tissue of the male patient was stored in the RNA Later solution (Thermo Fisher scientific, AM7020, Waltham, MA, USA). Total RNA was extracted using Trizol reagent (Takara, 9109, Tokyo, Japan) and cDNA was synthesized from total RNA using PrimerScript (Takara, RR047A). *KASH5* cDNA was amplified by PCR using the 2x Phanta Flash Mix (Vazyme, P515-01, Nanjing, China) with the following condition: 98°C 10 s, 55°C 3 s and 72°C 10 s for 38 cycles. The information of primers is available in **Supplementary Table 2**.

### Western blot

To obtain protein lysate, the testicular tissue was lyzed with lysis buffer [50 mM Tris-HCl (pH 7.5), 150 mM NaCl, 2.5 mM EDTA, and 0.5% Triton X-100], followed by centrifugation at 4°C for 15 min. The supernatant was collected and denatured for 10 mins in protein loading buffer [100mM Tris-HCl (pH 7.4), 2% SDS, 15% glycerol, 0.1% bromophenol blue, and 5 mM dithiothreitol (DTT)]. After SDS-PAGE electrophoresis, the separated protein was transferred onto the nitrocellulose filter membrane (GE Healthcare, 10600002, CT, USA). The membrane was blocked in TBST solution [50 mM tris (pH 7.4), 150 mM NaCl and 0.1% Tween-20] containing 5% skimmed milk for 1 hour, followed by incubation with primary antibodies at 4°C overnight. The membrane was then washed three times with TBST solution for 10 min each time. After one-hour incubation with horseradish peroxidase-coupled secondary antibodies, the membrane was developed by chemiluminescent substrate (Thermo Fisher scientific, 34580) using the ImageQuant LAS 4000 Imaging System (GE Healthcare). The information of primary and secondary antibodies used and their dilutions are provided in **Supplementary Table 3**.

### Hematoxylin and eosin staining

Testicular tissues were fixed in Bouin’s solution and embedded in paraffin for sectioning. In brief, the tissue slides were deparaffinized in xylene for 20 mins and then rehydrated with gradient ethanol (100%, 100%, 90%, 80%, 70%, and 50%), followed by staining with hematoxylin for 5 min and eosin for 1 min. After sequential dehydration with 50%, 70%, 80%, 90%, and 100% ethanol and transparency in xylene for 5 min, the tissue sections were sealed with neutral resin. The images were captured *via* a Nikon ECLIPSE 80i microscope (Nikon Instruments, Tokyo, Japan) equipped with a DR-Ri1 camera and processed with NIS-elements Basic Research software (Nikon Instruments).

### Plasmid construction

To investigate the effect of the *KASH5* frameshift mutation (c.1271_1274del), we constructed pEGFP-N1 plasmids expressing green fluorescent protein (GFP)-tagged wild-type SUN1, FLAG-tagged wild-type, and FLAG-tagged mutant KASH5. The coding sequences of *SUN1* and *KASH5* were amplified from human testis cDNA by PCR. Then the PCR products were ligated with pEGFP-N1 backbones. Both wild-type and mutant plasmids were verified by Sanger sequencing. Primers for plasmids construction are listed in **Supplementary Table 2**.

### Cell culture and transfection

HEK293T cells (human embryonic kidney 293 cells which contains the SV40 T-antigen, ATCC, CRL-3216) were cultured in high-glucose Dulbecco’s Modified Eagle’s Medium (DMEM) supplemented with 10% FBS (GIBCO, 16000-044, Carlsbad, CA, USA), 100 U/ml penicillin, and 100 mg/ml streptomycin (GIBCO, 15140-122), and maintained at 5% CO_2_. Cells were passaged 2-3 times after thawing and transfected at 70-80% confluency. Transfection of plasmids was performed using lipofectamine 3000 (Invitrogen L3000015, CA, USA).

### Immunofluorescence staining

The testicular tissue was fixed in 4% PFA and embedded in paraffin for sectioning. For immunofluorescence staining of slides, sections were kept at 65°C for 20 min and then dewaxed with xylene, followed by rehydration with gradient alcohol (100%, 90%, 80%, 70%, and 50%; 5 min each). After permeabilization in PBST [50 mM HCl (pH 7.4), 137 mM NaCl, 2.7 mM KCl, 4.3 mM Na_2_HPO_4_.7H_2_O, 1.4 mM KH_2_PO_4_ and 0.25% Triton-X100] for 1 h, the slides were boiled in citrate buffer (1.5 g 12-hydrated trisodium citrate and 0.2 g 1-hydrate citric acid dissolved in 500 ml ultrapure water) for antigen retrieval. Subsequently, sections were blocked with antibody dilution buffer (10% normal donkey serum, 3% bovine serum albumin [BSA], and 0.05% Triton X-100 in PBS) for 30 mins, washed with TBST solution, and then incubated with anti-KASH5 antibody (Thermo Fisher scientific, PA554116) for 12 h at 4°C. After washing with TBST three times, the slides were incubated with secondary antibodies at 37°C for 1.5 h, followed by another three washes with TBST. Lastly, the sections were air-dried and mounted in VECTASHIELD mounting medium (Vector Laboratories, H-1000, CA, USA) containing Hoechst 33342 (Invitrogen, H3570). For immunostaining of cultured cells, round glass slides were placed in the 24-well-plate prior to seeding cells for transfection. The slides were permeabilized with PBST followed by antibody incubation.

Images were captured using an Olympus BX61 microscope (Tokyo, Japan) equipped with a CCD camera (QICAM Fast 1394, QImaging, Burnaby, BC, Canada) and processed with Image-Pro Plus software (Media Cybernetic, Rockville, MD, USA). The information of primary and secondary antibodies used and their dilutions are provided in **Supplementary Table 3**.

### Co-immunoprecipitation

Plasmids expressing GFP-tagged wild-type SUN1 with FLAG-tagged wild-type or mutant KASH5 were co-transfected into HEK293T cells. Forty-eight hours post transfection, proteins were extracted from harvested cells using co-immunoprecipitation (co-IP) buffer (50mM Tris-HCL pH 7.5, 150mM NaCl, 0.5% TritonX-100, and 2.5 mM EDTA) supplemented with 1 mM PMSF (Thermo Fisher Scientific, 36978). Subsequently, proteins were incubated with anti-DYKDDDDK G1 Afinity Resin (GenScript, L00432-25, Nanjing, China) under gentle rotation at 4°C for 8 h. After washing with co-IP buffer six times, the co-IPed protein complexes were eluted in protein loading buffer and then denatured for 10 min. Finally, the protein complexes were detected *via* western blotting analysis. The information of primary and secondary antibodies used and their dilutions are provided in **Supplementary Table 3**.

## Results

### Clinical characteristics of patients

**Figure 1:**
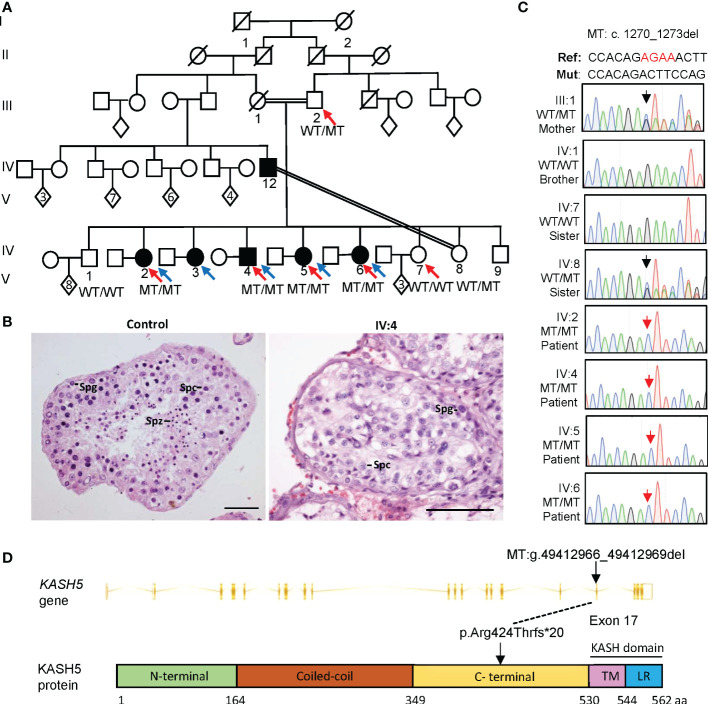
A homozygous *KASH5* mutation was identified from Pakistani patients suffering from reproductive failure and born to a consanguineous marriage. **(A)** The pedigree of the family. Squares represent males, circles represent females, diamonds indicate offspring with the numbers inside indicating the number of children, and the slash symbols denote deceased family members. Solid squares and circles indicate affected male and female patients, respectively. Parallel slash lines indicate consanguineous marriages. Red arrows indicate members receiving whole exome sequencing. Blue arrows indicate probands. **(B)** Representative images of H&E-stained testicular sections from a control man diagnosed with obstructive azoospermia and patient IV:4. Spg, spermatogonia; Spc, spermatocyte; Spz, spermatozoon. Scale bars denote 50 μm. **(C)** Sequencing chromatograms of peripheral blood gDNA from the patients and their father (III:2), brother (IV:1) and sisters (IV:7 and IV:8). The red arrow indicates the *KASH5* mutation (c.1270_1273del). **(D)** The gene structure and protein domains of KASH5 (Ensembl transcript ID: ENST00000447857.8). Vertical bars indicate exons and slashed lines represent introns. The non-mutant KASH5 protein (562 amino acids) comprises five domains: N-terminal domain, coiled-coil domain, the C-terminal domain, transmembrane (TM) domain, and the leucine-rich (LR) domain. The mutation (g.49412966_49412969del) locates in exon 17, which causes a frameshift within the C-terminal domain. Lines indicate the mutation positions at the genomic and protein levels (predicted).

A consanguineous Pakistani family with five siblings suffering from idiopathic reproductive failure was investigated in this study. The parents (III:1 and III:2) were first-degree cousins and gave birth to six daughters (IV:2, IV:3, IV:5, IV:6, IV:7 and IV:8) and three sons (IV:1, IV:4 and IV:9) (**Figure 1A**).

All daughters had a normal onset of puberty with no history of injury or infection of reproductive systems. IV:7 (34-yr-old) has been married for 15 years and gave birth to three children. IV:8 (32-yr-old) has been married for 10 years, but has not been conceived, and her husband was diagnosed as azoospermia, suggesting that the infertility was more likely attributed to the male factor. IV:2 (44-yr-old), IV:3 (42-yr-old), and IV:6 (37-yr-old) have been married for 23, 21, and 16 years, respectively, but all are childless despite trying to conceive. They were pregnant within the first year of marriage, but experienced recurrent miscarriage (4, 4, and 3 times for IV:2, IV:3, and IV:6 respectively) occurring in the 3rd month of pregnancy of unknown cause (**Table 1**). IV:5 (39-yr-old) has been married for 18 years and tried to conceive, but has never been pregnant. IV:2 and IV:3 entered menopause at the age of 41, and at present, IV:5 and IV:6 still have a normal menstrual cycle of 28-30 days, lasting for about seven days each time. Serum hormone measurements of IV:2, IV:5, and IV:6 detected elevated FSH levels (>12.5 mIU/mL) and declined AMH levels (<0.1 ng/mL) (**Table 1**). The level of LH (reference range: 2.4-12.6 mIU/ml) was 40 in IV:2, and was normal but close to the upper boundary of the reference range in IV:5 and IV:6 (**Table 1**). IV:5 claimed to have pelvic ultrasonography performed when she was 35-yr-old, and a dominant follicle was seen in one ovary. IV:6 had transvaginal ultrasonography performed at 36-yr-old on the 10th day of menstrual cycle. The results showed that the sizes of uterus and ovaries were normal, and antral follicles were visible but no dominant follicle observed (**Table 1**). The hormone and ultrasound results suggest that the female patients had DOR.

**Table 1:** Clinical characteristics of the female patients.

		IV:2	IV:3	IV:5	IV:6
Age (yr old)^a^	–	44	42	39	37
Age at marriage (yr old)	–	21	21	21	21
Karyotype	–	46, XX	46, XX	46, XX	46, XX
Time of the first pregnancy	–	The first year of marriage	The first year of marriage	–	The first year of marriage
Number of miscarriages	–	4	4	0	3
Diagnosis	–	Recurrent miscarriage	Recurrent miscarriage	Infertility	Recurrent miscarriage
Ultrasound ^b^					
Age (yr old) at the examination		–	–	35	36
Ovaries (mL)	>3.5	–	–	–	Left: 9.3;Right: normal
Dominant follicle number	–	–	–	A dominant follicle in one ovary	Left: nill Right: nill
Antral follicle number	–	–	–	–	Left: 1 Right: 2~3
Information of menstruation					
Age (yr old) of menarche	–	15	14	13	14
Menstrual cycle (days)	–	28-30	28-30	28	28
Duration of period (days)	–	7	7	7	7
Blood flow per cycle	–	normal flow	normal flow	normal flow	normal flow
Cycle information	–	menopause at 41 yr old	menopause at 41 yr old	regular	regular
Hormone concentrations ^b^					
Age (yr old) at the examination		44	–	39	37
FSH (mIU/mL)	3.5-12.5	65.6	–	12.7	26.3
LH (mIU/mL)	2.4-12.6	40.3	–	11.0	11.9
Prolactin (ng/mL)	4.8-23.3	9.8	–	4.8	2.5
Estradiol (pg/mL)	12.5-166	19.7	–	48.2	33.8
Progesterone (ng/mL)	0.2-1.5	0.1	–	1.6	0.2
AMH (ng/mL)	–	<0.03	–	0.06	0.03

a Age at manuscript submission.

b Reference values were suggested by local clinics.

LH: Luteinizing hormone; FSH: Follicle-stimulating hormone; AMH: Anti Mullerian Hormone.

All three brothers had a normal onset of puberty with no history of testicular injury or infection. IV:1 (46-yr-old) has been married for 22 years and has eight children, IV:4 (41-yr-old) has been married for 19 years, but suffered from infertility, and IV:9 (29-yr-old) was single and unmarried. IV:4 had two semen analyses conducted following the WHO guidelines, and both examinations showed that he had normal semen volume but no sperm seen in the ejaculates (**Table 2**). Sexual hormonal analyses revealed that he has reduced testosterone, but the levels of FSH, LH, prolactin and estradiol were all within the normal ranges (**Table 2**). To detect whether sperm exists in the testis of IV:4, testicular biopsy was conducted. Hematoxylin and eosin (H&E) staining of testicular sections revealed the presence of many spermatogonia and primary spermatocytes but a complete absence of spermatids and sperm in seminiferous tubules of IV:4 (**Figure 1B**). Immunofluorescence staining on testicular sections for the acrosome marker, PNA, and the metaphase cell marker, H3S10p (28, 29) confirmed the spermatogenesis arrest prior to meiotic metaphase I (**Supplementary Figure 2**). These results indicated that the male patient suffered from NOA and his spermatogenesis was arrested at the spermatocyte stage.

**Table 2:** Clinical characteristics of the male patient.

	Reference values	IV:4
Age (yr old) ^a^	–	41
Age (yr old) at marriage	–	22
Karyotype	–	46, XY
Diagnosis	–	NOA
Semen analysis ^b^		
Semen volume (mL)	>1.5	2.8 ± 1.1
Sperm count (millions/mL)	>15	0
Testis size (mL) ^c^		
Right testis	>12.5	17.9
Left testis	>12.5	14.0
Hormone concentrations ^c^		
FSH (mIU/mL)	1.5-12.4	10.7
LH (mIU/mL)	1.7-8.6	6.8
Prolactin (ng/mL)	4.04-15.2	2.4
Estradiol (pg/mL)	7.63-42.6	23.6
Testosterone (ng/mL)	300-890	208

a Age at manuscript submission.

b Reference values were published by WHO in 2021.

c Reference values were suggested by local clinics.

LH: Luteinizing hormone; FSH: Follicle-stimulating hormone.

### A homozygous *KASH5* frameshift mutation identified in patients

We performed whole exome sequencing (WES) on patients IV:2, IV:4, IV:5, and IV:6 (patient IV:3 refused to participate in our following research), as well as their sister IV:7 and father III:2. Variants were filtered following a series of criteria (**Supplementary Figure 1A**; **Supplementary Table 1**, described in details in materials and methods). Briefly, variants recessively co-segregating with infertility/recurrent miscarriage and variants dominantly segregating with female infertility and/or recurrent miscarriage were considered in priority. Among the retained variants, a frameshift mutation in *KASH5* was identified recessively segregating with the infertility and recurrent miscarriage within the family; a missense variant in *IZUMO1R* was found heterozygous in the female patients and their father, but not present in the fertile sister. IZUMO1R is implicated in fertilization and barely expressed after fertilization (30, 31), and *Kash5* mutant mice exhibit male and female infertility due to meiotic defects (as summarized in **Supplementary Table 1**). Considering that all our patients do not present other symptoms except infertility/recurrent miscarriage, the *KASH5* variant was thus identified as the variant most likely pathogenic for the reproductive defects of this family.

This *KASH5* frameshift mutation (Chr19: g. 49412966_49412969del; ENST00000447857.8, c.1270_1273del) has extremely low allele frequencies in the 1000 genomes, ExAC and GnomAD database (**Supplementary Table 1**). Furthermore, it is located in the linkage region and the RoH region, supporting its pathogenicity from the genetic perspective (**Supplementary Figure 1B, C**). Moreover, the *KASH5* gene is highly expressed in the testis, and known phenotypes of the *Kash5* knockout mouse model recorded in the FertilityOnline database are infertility in both sexes (32) (**Supplementary Table 1**). Further through Sanger sequencing of peripheral blood genomic DNA, we verified that the *KASH5* mutation was heterozygous in the father (III:1), homozygous in all patients participating in the sequencing analyses (IV:2, IV:4, IV:5 and IV:6), and not present in the fertile siblings (IV:1 and IV:7) (**Figure 1C**).

### Absence of KASH5 proteins and meiotic arrest prior to pachytene in the male patient

**Figure 2:**
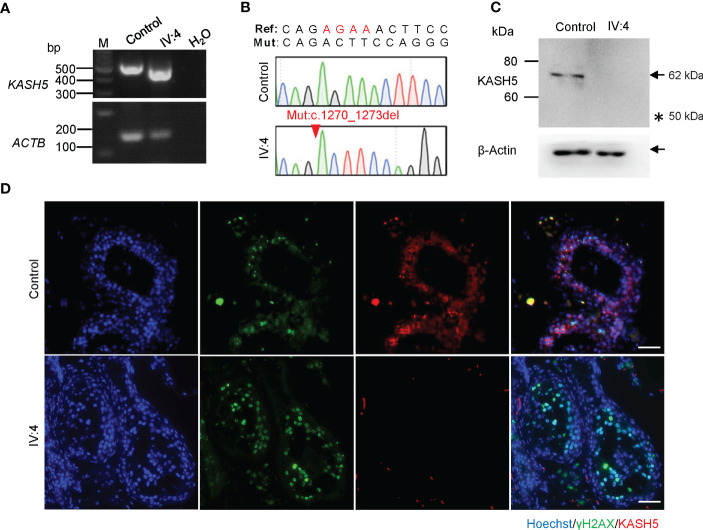
Absence of KASH5 proteins and meiotic arrest prior to pachytene stage in the male patient. **(A)** RT-PCR analysis of testicular samples from a man with obstructive azoospermia (serving as the control) and IV:4. *ACTB* served as the loading control. **(B)** Sequencing chromatograms of testicular cDNAs from the patient and control. The red arrow indicates the *KASH5* mutation (c.1270_1273del). **(C)** Analysis of the expression of KASH5 in the testicular sample of IV:4 by western blotting. β-Actin served as a loading control. Arrows indicate the bands corresponding the protein of interest and the asterisk indicates the position of the predicted truncated protein. **(D)** Immunofluorescence staining of testicular sections for KASH5 (red) and γH2AX (green). The nuclei were stained with Hoechst 33342 (blue). Scale bars, 50 µm.

The *KASH5* mutation (c.1270_1273del) is located in the exon 17 and introduces a premature stop codon, likely leading to the production of a truncated protein (p. Arg424Thrfs*20) or nonsense-mediated mRNA decay (**Figure 1D**). Reverse-transcription analyses of the testicular cDNA revealed a similar level of *KASH5* mRNA in the patient to that in the control man who was diagnosed with obstructive azoospermia (**Figures 2A, B**). To detect the effect of the mutation on KASH5 protein expression, we performed western blotting using the anti-KASH5 antibodies raised against an antigen of 113-187 amino acids of human KASH5 protein. No band corresponding to the full-length KASH5 protein or the predicted truncated protein was seen in testis of the patient (**Figure 2C**). These results indicate that the *KASH5* frameshift mutation leads to an absence of full-length or truncated KASH5 protein in the testis of patient.

To examine the exact stage of spermatocyte arrest in the patient, we performed immunofluorescence staining for γH2AX, which marks DSBs or sex bodies and is often used to distinguish early or late spermatocytes based on its staining pattern(33). As shown in **Figure 2D**, in the seminiferous tubules of the control, we found many cells showing pan-nuclear or dotted γH2AX signals, which corresponds to typical leptotene/zygotene and pachytene/diplotene spermatocytes, respectively. However, in the patient, only cells labeled with pan-nuclear signals were seen in tubules, indicating that the meiosis arrests prior to the pachytene stage with massive DSBs unrepaired. In addition, immunofluorescence staining also revealed that, in the seminiferous tubules of the control man, KASH5 signals localizing on the nuclear envelope were seen in spermatocytes as expected, but no KASH5 signals were detected in the spermatocytes with γH2AX signals (**Figure 2D**). Together, these results indicate that the absence of KASH5 proteins and meiotic arrest prior to pachytene in testicular cells of the male patient.

### Diminished interaction between the predicted truncated KASH5 protein and SUN1

Among the four affected sisters, three of the infertile sisters suffered from recurrent miscarriage, indicating that mature follicles were formed and fertilization occurred (34) for them; IV:6 had normal sizes of ovaries, normal menstrual cycles, and visible antral follicles even at her mid-30s (**Table 1**). This is very surprising, as the ovaries were barely visible with no identifiable follicles in the female adult *Kash5*-null mice (13). In the previous reports, all the three women reported with *KASH5* mutations, which abolished the nuclear envelope distribution of the LINC complex, presented typical POI phenotype at their 20s or early 30s, including FSH levels higher than 40 mIU/mL, oligomenorrhea that ceased at 27, 31, and 23 years old, respectively, and small ovaries without visible follicles (16, 18). Given that meiotic checkpoints exhibit sexual dimorphism, meiocytes with unrepaired DSBs and unpaired homologous chromosomes, which were monitored and cleared at pachytene in males, but could undergo folliculogenesis and generate eggs with an abnormal number of chromosomes in females (35, 36). Thus, we suspected that the KASH5 truncated protein retaining some function may be expressed in ovaries of patients, which allows the production of eggs with an abnormal number of chromosomes and fertilization, but the fetus consequently ended in spontaneous abortion due to aneuploidy. However, due to the unavailability of fetal ovarian tissues and KASH5 is hardly expressed in human adult ovaries (32, 37), we are not able to test this conjecture in our patients.

**Figure 3:**
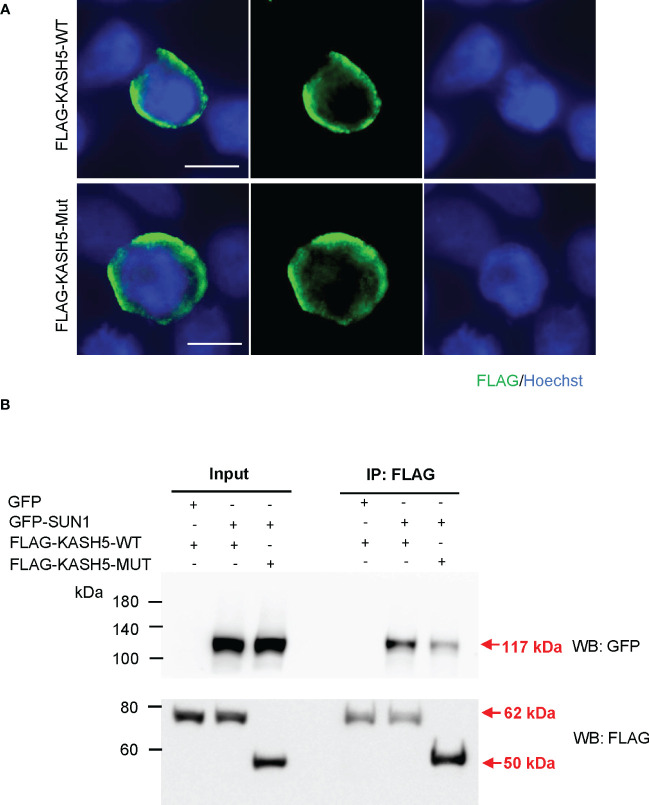
Diminished interaction between the predicted truncated KASH5 and SUN1. **(A)** Immunofluorescence staining using the anti-FLAG antibodies in HEK293T cells transfected with plasmids expressing FLAG-KASH5-WT or FLAG-KASH5-MUT proteins. Scale bars, 10 µm. **(B)** Co-immunoprecipitation using the anti-FLAG antibody with HEK293T cells transfected with plasmids expressing GFP-SUN1 proteins and plasmids expressing FLAG-KASH5-WT or FLAG-KASH5-MUT proteins, followed by western blotting analyses using anti-GFP or anti-FLAG antibodies. Cells transfected with GFP-empty plasmids serves as a negative co-IP control. Arrows indicate the bands corresponding to the proteins of interest.

As an alternative choice, we attempted to examine the expression, localization, and function of the predicted truncated KASH5 proteins *in vitro*, by transforming the plasmids expressing N-terminal FLAG-fused with wild-type or with truncated KASH5 into HEK293T cells. Immunofluorescence staining and western blotting showed that the truncated KASH5 proteins could be expressed in cultured cells and showed circular localization around nuclei, similarly to the localization of wild-type KASH5 proteins (**Figure 3A**). Furthermore, co-IP were performed in HEK293T cells co-expressing N-terminal GFP-fused SUN1 proteins with the FLAG-tagged wild-type or truncated KASH5 proteins. Surprisingly, we found that the truncated KASH5 proteins could still interact with SUN1, though the interaction was much weaker than that of the wild-type KASH5 (**Figure 3B**). Collectively, these findings suggest that the predicted truncated KASH5 protein could localize on the nuclear envelope and showed a reduced interaction with SUN1, which may be sufficient to avoid oocyte elimination due to meiotic abnormalities in prophase and support normal follicular development and egg generation.

## Discussion

Here, we identified a frameshift mutation in *KASH5* (c.1270_1273del, p. Arg424Thrfs*20) in a consanguineous Pakistani family and found that this mutation contributes to human reproductive failure in both sexes with sexual dimorphism in germ cell development. This mutation leads to the absence of KASH5 protein in the testes, resulting in meiotic arrest prior to pachytene stage and NOA in the affected brother, which is in congruent with previous reports of KASH5-null men and male mice (13, 16, 17). However, different from the small ovaries and invisible follicles in women and female mice null for KASH5, the four affected sisters displayed DOR and three of them suffered from recurrent miscarriage, instead of POI. Given that the truncated KASH5 protein retains a weakened interaction with SUN1, we infer that the mutant KASH5 protein may be expressed, which is sufficient to avoid oocyte elimination due to meiotic abnormalities in prophase occurring in KASH5-null female mice and women, and support follicular development and aneuploid egg generation but consequently miscarriage in the female patients. In addition, we found that the reproductive defects are different among female siblings carrying the same homozygous *KASH5* mutation: IV:2, IV:3, and IV:6 had recurrent miscarriages within the third month of gestation, whereas IV:5 has never been pregnant. One possible explanation is that the poor-quality embryos of IV:5 may die much earlier, for example, prior to the implantation before pregnancy could be detected. We also could not rule out the possibility that, though no candidate infertility-causing mutations were identified in the WES data of IV:5, IV:5 harbor mutations non-coding regions or mutations in genes with uncharacterized functions, resulting in the infertility. These interesting and unexpected findings give an opportunity to deepen the understanding of meiosis regulation and gamete quality control in humans, especially women.

Sexual dimorphism in germ cell survival and development has been reported for mutations in meiotic proteins in humans and mice. For example, *Sycp3^-/-^* female mice showed subfertility and experienced loss of embryos resulting from aneuploid oocytes, while spermatogenesis arrest at the zygotene stage of meiosis I in the mutant males (38, 39); female mice or women mutant for MEI1 or TOP6BL retained oocytes at the adult age but were suggest to have oocyte maturation failure, while the affected men or mice displayed meiotic arrest at the zygotene stage (40–44). The phenomenon is partially explained by the sexual differences in the stringency of and responses to meiotic checkpoints, which could allow folliculogenesis and even fertilization in the female mutants (45). Notably, knockout of *Tex15, Zcwpw1, Zfp541*, *Kctd19*, or *M1ap*, etc., causing meiotic arrest at prophase or metaphase in male mice, does not affect fertility of females (46–51), suggesting molecular differences *per se* in meiotic regulation of oocytes and spermatocytes, in addition to checkpoints. Our study on the five siblings harboring the same *KASH5* mutation provide new evidence for sexual dimorphism for *KASH5* mutations.

The KASH domain, comprising of the C-terminal transmembrane domain and leucine-rich domain, is essential for the telomere localization and interaction with SUN1 in mice (12, 13). In our study, the truncated human KASH5 protein (p. Arg424Thrfs*20) lacks the entire KASH domain, but still possess the ability to localize to nuclear envelope and a weakened interaction with SUN1 when expressed in cultured cells, suggesting that the non-KASH domains of the human KASH5 protein also play a role in forming the LINC complex. These results also indicate that the mouse and human KASH5 proteins may have some differences. Besides, the observation that the predicted truncated KASH5 protein was expressed in cultured HEK293T cells but was not detected in the patient’s testis indicates that detection of protein expression in cultured cells after plasmids transfection may not reflect the situation *in vivo*. Such an intriguing phenomenon may be attributed to potentially tissue-specific post-translational modifications, protein folding, or protein half-lives, and further investigations are needed to unveil the underlying mechanism.

Another intriguing observation in our female patients is that their clinical manifestations (recurrent miscarriage and/or DOR) appear less severe than those of the female mice carrying a similar *Kash5* mutation (*Kash5^del/del^*) recently reported by Zhang et al. (18). In *Kash5^del/del^* mice, a 7-bp deletion was introduced in the exon 15 of *Kash5* gene and leads to a frameshift from amino acid position 467, near the mutation site (equivalent to amino acid position 505 in the mouse KASH5 protein) of our patients. The expression of the truncated mutant protein was unknown in *Kash5^del/del^* mice, but the oogenesis and spermatogenesis defects resemble those of the adult *Kash5^-/-^* mice (13). *Kash5^del/del^* female mice exhibited meiotic arrest at the zygotene stage, dramatic oocyte depletion at 3 dpp, and very small ovary with no oocytes detected at 21 dpp (18), in contrast to the observation of normal ovary size and regular menstrual cycles with folliculogenesis even at their mid-30s in our patients.

There are several potential explanations for the difference between our female patients and the *Kash5^del/del^* female mice. First, the predicted truncated KASH5 protein is present and retain some function in humans, but not in mice (as discussed above). Second, the critical function of KASH5 proteins may be compensated by some unknown proteins in women, but not in mice. Third, our patients may harbor mutations in some unknown genes functioning in meiosis or folliculogenesis checkpoints that permits follicle development. Forth, the phenotypic discrepancy in humans and mice may be attributed to potential differences in meiosis or folliculogenesis regulation in the two species. In our previous study, the female patient carrying a homozygous *TOP6BL* mutation (c.483dupT) showed primary infertility, with normal hormone levels, menstrual cycles and ovary sizes with follicular activity but no sign of DOR or POI at the age of 35 (43); recurrent hydatidiform moles or miscarriage were also reported for women carrying the same mutation in another study (52). However, the female *Top6bl* mutant mice showed very small ovaries and no oocytes detected by 20 weeks of age (43). In congruent with this, it is recently reported that two infertile sisters carrying a homozygous *DMC1* frameshift mutation showed a reduced antral follicle number but metaphase II oocytes could be retrieved, while the mutant mice carrying the same mutation showed total failure of follicle development at postnatal day 12 (53). Thus, our study along with the previous findings indicates an unknown mechanism that allows the folliculogenesis and fertilization in spite of the severe meiotic defects in the humans.

In summary, we have provided genetic and functional evidence that a homozygous loss-of-function mutation in *KASH5* leads to reproductive failure in both sexes with sexual dimorphism and bring forward KASH5 as a recurrent miscarriage-associated gene. These findings expand the clinical presentations associated with *KASH5* mutations and promote genetic counselling and appropriate guidance for NOA, DOR, and recurrent miscarriage. Moreover, these findings allow an in-depth understanding of sexual dimorphism in germ cell development, also shed a new perspective for the differences in ovarian follicular development and oogenesis between human and mouse, and provide a scientific basis for genetic counseling and diagnosis of human reproductive failure.

## Data availability statement

The original contributions presented in the study are included in the article/**Supplementary Material**. Further inquiries can be directed to the corresponding authors.

## Ethics statement

The studies involving human participants were reviewed and approved by institutional ethics committee of the University of Science and Technology of China (USTC). The patients/participants provided their written informed consent to participate in this study. Written informed consent was obtained from the individual(s) for the publication of any potentially identifiable images or data included in this article.

## Author contributions

XH performed the experiment and collected the data, investigation, validation, writing—original draft. AZ, BS, ZM, IK, QZ, and WS collected the human samples. SD collected the clinical information of female patients. JZ and HZ were involved in the whole-exome sequencing and screening for the mutations. LW and XJ was involved in the critical discussion. HM was involved in the study design, execution, and analysis, manuscript writing, review, and editing. QS was involved in the project administration and supervision, manuscript review, and editing. All authors contributed to this work, discussed the results, critically reviewed, and revised the manuscript. All authors contributed to the article and approved the submitted version.
