# Supplementary material for: A homozygous KASH5 frameshift mutation causes diminished ovarian reserve, recurrent miscarriage, and non-obstructive azoospermia in humans
Source: Front Endocrinol (Lausanne). 2023 Feb 14;14:1128362. doi: 10.3389/fendo.2023.1128362 (PMC9971600; doi:10.3389/fendo.2023.1128362)
Supplement: Supplementary file 1 [file DataSheet_1.docx]

Supplementary Material

**A homozygous KASH5 frameshift mutation causes diminished ovarian reserve, recurrent miscarriage, and non-obstructive azoospermia in humans**

Xiaoning Hou*, Aurang Zeb

***Correspondence:** Qinghua Shi: qshi@ustc.edu.cn (Q.S.); Hui Ma: clsmh@ustc.edu.cn (H.M.)

**Supplementary Material**

**
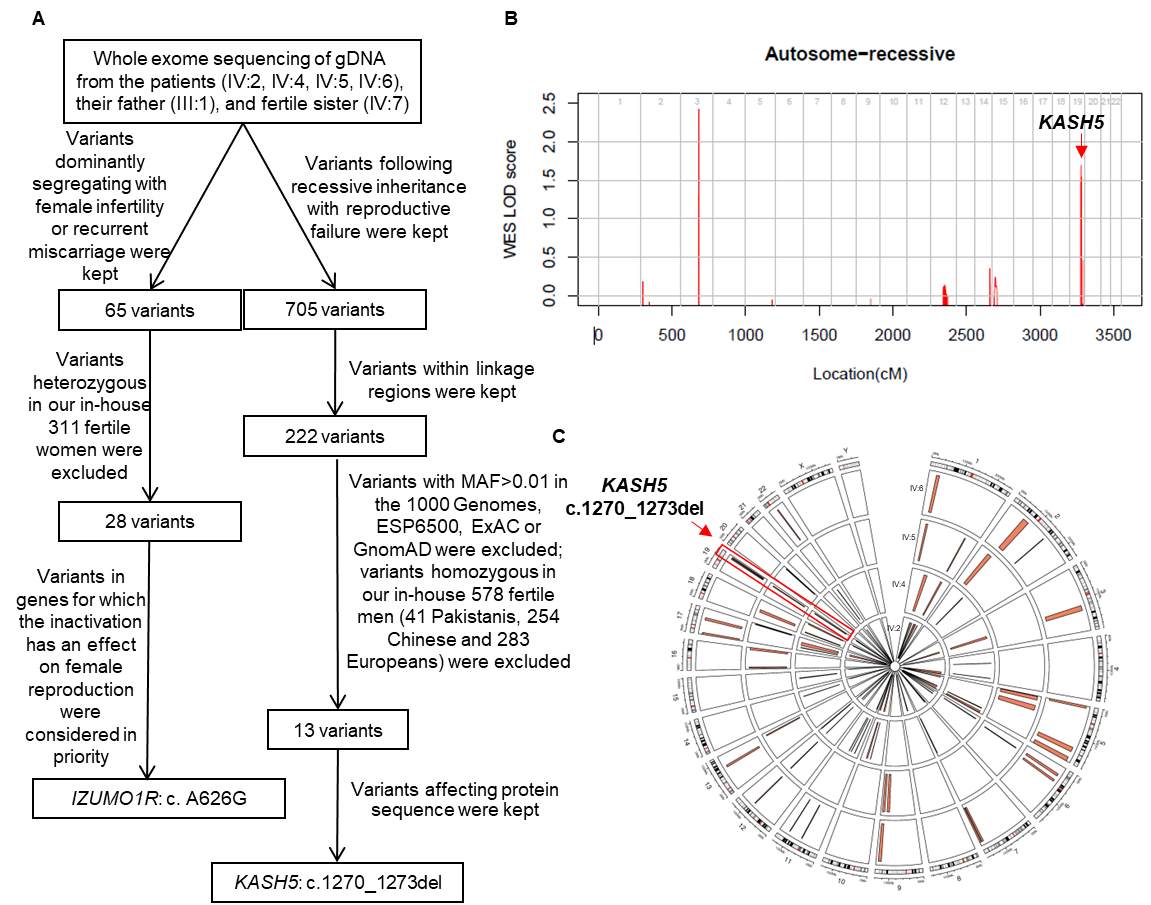
**

**Supplementary Figure 1. The workflow for the whole-exome sequencing data analysis.** (**A**) The WES data analysis pipeline. MAF, minor allele frequency. (**B**) Genome-wide logarithm of the odd scores (LOD) derived from the WES data. (**C**) Homozygosity mapping analysis for individuals carrying the *KASH5* mutation.

**
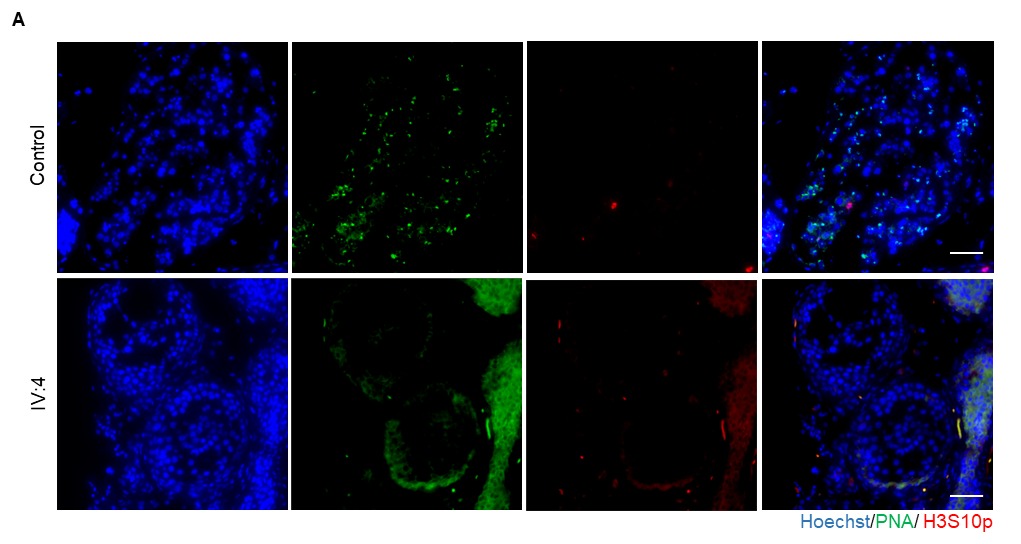
**

**Supplementary Figure 2. Spermatogenesis defects in the male patient.**

(A) Immunofluorescence staining of testicular sections for PNA (Green) and H3S10p (Red). The nuclei were stained with Hoechst 33342 (blue). Scale bars, 50 µm.

| **Gene symbol** | **Mutation type** | **cDNA alteration** | **Amino acid alteration** | **Genotype** | | | | | | **Remark** | **Reference** | **Allele frequency** | | | |
| --- | --- | --- | --- | --- | --- | --- | --- | --- | --- | --- | --- | --- | --- | --- | --- |
|  |  |  |  | **III:1 Father** | **IV:2 Female Patient** | **IV:4 Male patient** | **IV:5 Female patient** | **IV:6 Female patient** | **IV:7 Female patient** |  |  | **1000 Genomes** | **ExAC** | **gnomAD** | **ESP6500** |
| *KASH5* | frameshift substitution | 1270_1273G | Arg424Thrfs*20 | het | hom | hom | hom | hom | ref | *Kash5* knockout mice were infertile in both sexes. Spermatocytes failed in pairing of homologous chromosomes, and exhibited meiosis arrest in zygotene. Adult female knockout mice had very small ovaries with a complete absence of oocytes. | DOI:10.1083/jcb.201304004 | 0 | 0 | 0.000004065 | 0 |
| *IZUMO1R* | nonsynonymous SNV | A626G | K209R | het | het | het | het | het | ref | This gene, also known as *Juno*, encodes the receptor for IZUMO1 present at the cell surface of oocytes (oolemma). Female mice lacking Juno were infertile and Juno-deficient eggs did not fuse with normal sperm | DOI:10.1038/nature13203 | 0 | 0 | 0 | 0 |

**Supplementary Table 1. Variants identified by WES following bioinformatic** analysis

**Supplementary Table 2 Information of primers used in this study.**

| **Primer name** | **Sequence (5'- 3')** | **Product (bp)** |
| --- | --- | --- |
| GFP-h-SUN1-F | TCGAGCGGTGGAGCTGCAGGAATGGATTTTTCTCGGCTTCA | 2358 |
| GFP-h-SUN1-R | GATCTAGAGTCGCGGCCGCTTCACTTGACAGGTTCGCCAT |  |
| FLAG-h-KASH5-WT-F | GATTACAAAGACGATGACGATAAAGACCTGCCCGAGGGCCCGGT | 1689 |
| pCR3-h-KASH5-WT-R | GATCTAGAGTCGCGGCCGCTTCACACTGGAGGGGGCTGGA |  |
| h-KASH5-MUT-F | TGGGGGACAGACTTCCAGGG | 426 |
| pCR3-h-KASH5-MUT-R | GATCTAGAGTCGCGGCCGCTTCACACTGGAGGGGGCTGGA |  |
| FLAG-h-KASH5-MUT-F | GATTACAAAGACGATGACGATAAAGACCTGCCCGAGGGCCCGGT | 1279 |
| h-KASH5-MUT-R | CCCTGGAAGTCTGTCCCCCA |  |
| h-KASH5-cDNA-F | AGGAACTGAGGCTGGAGATT | 509 |
| h-KASH5-cDNA-R | GACTGGGACCAGCTTTTTCA |  |
| h-cDNA-*ACTB*-F | AATGAGCTGCGTGTGGCTC | 148 |
| h-cDNA-*ACTB*-R | ATAGCACAGCCTGGATAGCAAC |  |

**Supplementary Table 3 Information of antibodies used in this study.**

| **Primary antibodies** | |  |  |  |
| --- | --- | --- | --- | --- |
| Target | Dilution | Host species | Supplier | Catalog number |
| PNA | 1:100 | - | Life technologies | L32458 |
| H3S10p | 1:100 | Rabbit | Santa Cruz | Sc-8656-R |
| KASH5 | 1:100 | Rabbit | Thermofisher | PA554116 |
| β-Actin | 1:3000 | Rabbit | Abcam | ab8227 |
| γH2AX | 1:5000 | Mouse | Millipore | 05-636 |
| GFP | 1:3000 | Rabbit | DUONENG-BIO | AB010201 |
| FLAG | 1:3000 | Rabbit | Life technologies | 2368S |
| **Secondary antibodies** | |  |  |  |
| Target | Dilution | Host species | Supplier | Catalog number |
| Mouse (Alexa-488) | 1:100 | Goat | Molecular Probes | A-21121 |
| Rabbit (Alexa-555) | 1:300 | Donkey | Molecular Probes | A31572 |
| Rabbit (Alexa-488) | 1:300 | Donkey | Molecular Probes | A21206 |
| Mouse (HRP) | 1:10000 | Goat | Biolegend | 405306 |
| Rabbit (HRP) | 1:10000 | Donkey | Biolegend | 406401 |
